# MAIT Cells Come to the Rescue in Cancer Immunotherapy?

**DOI:** 10.3390/cancers12020413

**Published:** 2020-02-11

**Authors:** Zuzanna Lukasik, Dirk Elewaut, Koen Venken

**Affiliations:** 1 Department of Internal Medicine and Pediatrics (Rheumatology Unit), Faculty of Medicine and Health Sciences, Ghent University, Ghent 9000, Belgium; 2 Molecular Immunology and Inflammation Unit, VIB Center for Inflammation Research, Ghent 9052, Belgium

**Keywords:** MAIT, unconventional, T cells, immunotherapy

## Abstract

Recent progress in immunobiology has led to the observation that, among cells classically categorized as the typical representatives of the adaptive immune system, i.e., T cells, some possess the phenotype of innate cells. Invariant T cells are characterized by T cell receptors recognizing a limited range of non-peptide antigens, presented only in the context of particular molecules. Mucosal-associated invariant T cells (MAIT cells) are an example of such unconventional cells. In humans, they constitute between 1% and 8% of the peripheral blood T lymphocytes and are further enriched in mucosal tissues, mesenteric lymph nodes, and liver, where they can account for even 40% of all the T cells. MAIT cells recognize antigens in the context of major histocompatibility complex class I-related protein (MR1). Upon activation, they instantly release pro-inflammatory cytokines and mediate cytolytic function towards bacterially infected cells. As such, they have been a rapidly evolving research topic not only in the field of infectious diseases but also in the context of many chronic inflammatory diseases and, more recently, in immuno-oncology. Novel findings suggest that MAIT cells function could also be modulated by endogenous ligands and drugs, making them an attractive target for therapeutic approaches. In this review, we summarize the current understanding of MAIT cell biology, their role in health and disease and discuss their future potential in cancer immunotherapy. This is discussed through the prism of knowledge and experiences with invariant natural killer T cells (iNKT)—another prominent unconventional T cell subset that shares many features with MAIT cells.

## 1. Untangling the Riddle of MAIT Cells’ Biology

**Figure 1:**
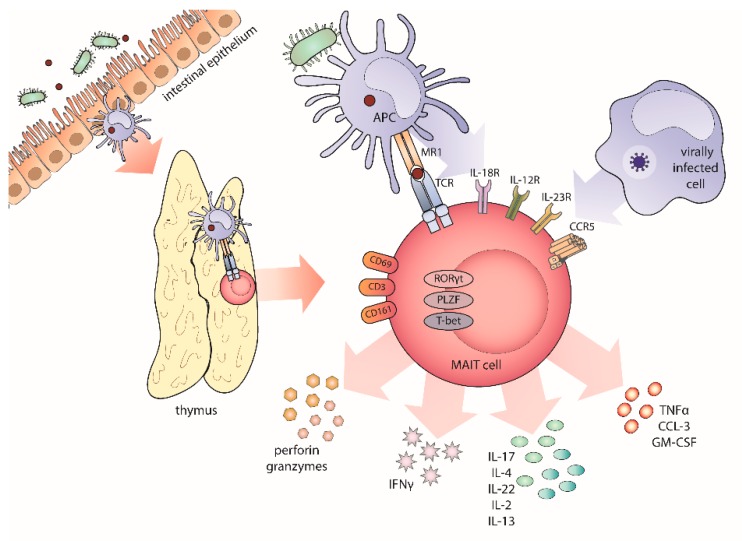
Mucosal-associated invariant T cells (MAIT) cell biology. MAIT cells are the largest antigen-specific T cell population in the human immune system. They recognize vitamin B-derived metabolites presented in the context of the major histocompatibility complex class I-like protein (MR1) molecule. MAIT cells develop in the thymus where they acquire tissue-homing properties in a three-stage process dependent on commensal microbiota and antigen-presenting cells. MAIT cells further mature in the periphery. Due to an array of cytokine and chemokine receptors, they respond to costimulatory signals provided by other immune cells and rapidly produce cytotoxic mediators, interferon gamma (IFNγ), tumor necrosis factor alpha (TNFα), and numerous interleukins. MAIT cells represent the innate-like branch of the immune system and, owing to their immediate, tailored-to-factor activation, orchestrate the immune response.

The characteristic feature of innate-like T cells are subtype-specific semi-invariant T-cell receptors (TCR) interacting with particular antigen-presenting molecules and recognizing non-peptide antigens. Mucosal-associated invariant T cells (MAIT) cells display a semi-invariant α-chain of the TCR (Vα7.2-Jα33/20/12 in humans and Vα19 in mice) combined with a limited repertoire of TCR-β chains, predominantly from TRBV6 and TRBV20 gene families [1] (Figure 1). Their antigen recognition is restricted to non-peptide molecules presented in the context of non-polymorphic major histocompatibility complex (MHC) class I-like protein MR1 [2]. These antigens include vitamin B metabolites synthetized by a range of microorganisms. Vitamin B metabolism pathways are highly conserved across species, and therefore antigen presentation in the context of MR1 molecule allows for the recognition of a group of pathogens upon their shared metabolic signature [3]. Furthermore, MR1 restricted antigens are bound and presented without host–cell processing [4]. Several immune cell types, including monocytes, dendritic cells, and B cells express MR1 molecules. The default extracellular expression of MR1 is very low—in the absence of their ligands, MR1 molecules are mostly maintained in an unfolded conformation in the endoplasmic reticulum [5,6]. Antigen binding results in a transient relocation of MR1 molecules to the cell surface, as, within hours from antigen presentation, they become internalized again [7]. Moreover, surface expression of MR1 is upregulated under inflammatory conditions. Interestingly, functional MR1 expression was found on several cancer cell types [8,9].

This observation is particularly interesting in the context of thymic MAIT cell development. It was shown that MAIT cells are selected by interaction with MR1-expressing thymocytes but not thymic epithelial cells nor thymus-resident immune cells [1,10]. The selection is maintained in germ-free bred mice, suggesting possible existence of endogenous MR1 ligands [2]. MAIT cells egress from thymus displaying a naïve phenotype and as such are present in low numbers in cord blood. After birth, they acquire a memory, an innate-like phenotype, and expand into the peripheral blood and the mucosal tissues. This process is dependent on a timely sequence of events: intra-thymic induction of a tissue-residency program [11], commensal flora colonization [2], and priming by MR1-expressing antigen-presenting cells [12]. In the absence of commensal microbes and in B-cell deficient subjects, naïve MAIT cells fail to mature and expand, and the entire population diminishes [1]. These findings on MAIT cells’ peripheral maturation and expansion are further supported by the fact that these cells are more abundantly present in the peripheral blood than in the thymus at any age of individual development. The number of circulating MAIT cells increases steadily in the first three decades of life and then decreases gradually [13]. Knowledge on age-dependent fluctuations in MAIT cell frequencies in mucosal tissues is currently lacking.

MAIT cells were first discovered and characterized as a double negative (DN) CD4-CD8- T cell population abundant in the peripheral blood of healthy subjects and expressing an invariant TCR α chain and the CD161 surface marker [14]. These markers initially served for identification and further phenotyping of human MAIT cells. From these studies, it was clear that MAIT cells express CD26, CD44, CD69, and CD25 surface molecules as well as cytokine receptors such as interleukin 7 receptor (IL-7R), IL-12R, IL-15R, and IL-18R and transcription factors PLZF (promyelocytic leukaemia zinc finger), RORγt (RAR-related orphan receptor gamma), and T-bet (T-box transcription factor) [15]. Closer analysis of the expression of CD4 and CD8 co-receptors on human peripheral blood MAIT cells revealed that, in fact, the majority of them were CD8+CD4-. Only about 14% of the peripheral blood MAIT cells were double-negative. In addition, a minor subset of these cells was described as CD4+CD8− single positive or CD4+CD8+ double-positive [16]. It was revealed that these subsets differentially decline with age, suggesting further variances in the regulation of their development [16]. The functional role of CD4 and CD8 co-receptors in MAIT cells biology remains to be elucidated, but distinct patterns of cytokine production were noticed between the subsets [17]. Studies in mice showed clear polarization between IL-17A- and interferon y (IFNγ)-producing sub-lineages, expressing transcription factors RORγt and T-bet, respectively. MAIT17 development was recently found to be strongly dependent on TCR signals received in the thymus, with this subset being hugely decreased in germ-free mice. Upon colonization, thymic expansion and maturation of MAIT17 cells was observed, with no changes in MAIT1 cell numbers [18]. The latest research confirms that the bifurcation into MAIT1 and MAIT17 subtypes occurs within the thymus and reveals further factors contributing to MAIT cell maturation and differentiation [19,20]. In contrast to mice, no clear partition into MAIT1 and MAIT17 subtypes is observed in human MAIT cells, which are also potent producers of tumor necrosis factor α (TNFα).

Functional studies revealed that different MR1 ligands may induce distinct effects on MAIT cells. Riboflavin (vitamin B2)-derivatives were observed to potently activate MAIT cells [21], while folate (vitamin B9)-derived antigens do not seem to do so. Riboflavin derivatives bound by MR1 are pyrimidine adducts of its unstable precursor, 5-Amino-6-D-Ribitylaminouraci (5-A-RU). These compounds are generated by most of the Prokaryotes and Fungi but not in animals devoid of the riboflavin biosynthesis pathway [22]. Interestingly, certain pathogenic bacteria have lost the ability to synthesize riboflavin and have adapted to its exogenous uptake. These facts partially explain the dependence of MAIT cell development on timely commensal colonization and the changes in MAIT cell activity observed in dysbiosis [23]. A small percentage of peripheral blood MAIT cells was discovered to be reactive towards the MR1 molecule itself [16]. The introduction of MR1-Ag tetramers [loaded with 5-(2-oxoethylideneamino)-6-D-ribitylaminouracil (5-OP-RU)] into the MAIT cell research was a breakthrough allowing for the recognition of distinct phenotypical and functional MAIT cell subsets [24,25]. Since human MAIT cells were initially identified upon TCRVα7.2 and CD161 markers, the abundance and the specific physiology of the described subsets in mucosal tissues and liver need further research by use of MR1 tetramer staining methods.

Importantly, MAIT cells can be activated independently of their TCR stimulation. Due to their high expression of several cytokine and chemokine receptors (IL-18R and IL-12R in particular), they respond to mediators released by other immune cells during infection. The protective role of MAIT cells in viral infections is completely MR1 independent and mostly relies on IL-18 signaling (in synergy with other cytokines) [26]. Type I interferons provide a strong costimulatory signal, enhancing MAIT cell secretory activity [27]. The MAIT cell response to pathogens is intensified and fine-tuned by the type of antigen-presenting cells involved, immune checkpoints signaling and cytokine stimulation additional to MR1-bound antigen recognition [28]. Moreover, these costimulatory signals enable MAIT cell proliferation at the site of infection, which is not induced by TCR stimulation alone [29]. Inducible T-cell costimulator (ICOS), which is constitutively highly expressed on the surface of MAIT cells, and IL-23 were recently identified as crucial second signals [28].

Upon activation, MAIT cells robustly release perforin and granzymes and produce the pro-inflammatory cytokines TNFα, IFNγ, and IL-17A as well as the potent granulocyte stimulation factor MIP-1. Production of other cytokines such as IL-22, IL-13, IL-26, or IL-2 was described in the context of different cytokine milieus and tissue subsets [29,30,31,32]. The rapid MAIT cell response to pathogenic bacteria is fully MR1-dependent [33], but the long-range activation is shaped by the combination and the amount of other stimuli [34,35]. Interestingly, IL-12 and IL-18 stimulation resulted in a dominant IFNγ production, while MR1 signaling was related to TNFα and IL-17A [33]. IL-7 triggered local proliferation and tissue accumulation of different IL-17-producing innate-like T cells, including MAIT cells [36]. The release of the aforementioned mediators results in destruction of infected cells and activation of other immune cell types [37]. This includes stimulation of dendritic cell maturation, which consequently leads to mobilization of natural killer (NK) cells and conventional T cells and evokes a cascade of immune events [38]. Recent transcriptomic analyses suggested that the role of MAIT cells in mucosal immunology expands beyond pathogenic microbes detection and also covers barrier maintenance [29,30]. MAIT cells exhibit transcriptomic signature associated with epithelial barrier integrity preservation, which was further upregulated during acute infection [29]. In-vitro stimulation of human MAIT cells with 5-OP-RU, akin to mouse MAIT cell activation by experimental bacterial infection (*Legionella*), resulted in striking upregulation of not only pro-inflammatory gene sets but also genes related to tissue repair (transforming growth factor beta-1, platelet derived growth factor subunit B and matrix metallopeptidase 25) and angiogenesis (granulocyte-macrophage colony-stimulating factor, vascular endothelial growth factor, hypoxia inducible factor 1 subunit alpha) [29]. This finding is in line with increased gut permeability in Mr1 knockout mice [39] and was confirmed on a functional level by wound-healing assay experiments [30,40].

## 2. The Harmony of Invariant T Cells

Studies on MAIT cell development and effector functions point towards their close relationship with another unconventional T cell population, the invariant natural killer T cells [41] (Table 1). Invariant natural killer T cells (iNKT) cells express a semi-invariant αβTCR (human Vα24/Jα18 combined with Vβ11, mice Vα14–Jα18 combined with Vβ2, Vβ7, or Vβ8.2), which allows them to recognize lipid antigens presented by CD1d molecules [42]. Similar to MAIT cells, iNKT cells are capable of secreting large amounts of cytokines and chemokines upon activation in a TCR-dependent or a TCR-independent way [43]. Moreover, both unconventional T cell populations (and their subsets) share the expression of transcription factors T-bet, RORγt, and PLZF [44]. The expression of T-bet and RORγt is required for IFN-γ and IL-17 production, respectively. PLZF was found to regulate cell effector maturation and orchestrate expansion in non-lymphoid tissues, the characteristic feature of MAIT and iNKT cells [45]. Mucosal surfaces allow for further maturation and phenotype acquisition of both innate-like cell populations. In contrast to MAIT cells, however, the intrathymic development of iNKT cells is irrespective of commensal microbiota, as no changes in iNKT numbers are seen in germ-free bred mice [18]. Nevertheless, common gene products were found crucial for their intrathymic development, such as members of the signalling lymphocyte activation molecule (SLAM) family receptors [19]. This might reflect a developmental competition between MAIT and iNKT cells or/and indicate that the two invariant T cell populations share complementary immune functions. In line with this, it has been shown that CD1d-deficient (NKT cell-deficient) mice exhibit increased numbers of MAIT cells (and vice versa) [2]. However, despite vast interindividual differences in MAIT and iNKT cell abundance, the ratio of the two populations across human blood samples seems to be constant in health (and sometimes disease), suggesting they are also controlled by similar genetic and/or environmental factors, at least in the periphery [41,46,47]. Altogether, the multi-level resemblance leads to the idea that MAIT and iNKT cells are complementarily bridging innate and adaptive immunity, and this interdependence is tightly regulated by common developmental or homeostatic factors while retaining their specific role in non-peptide TCR mediated immune responses.

## 3. MAIT Cells in Immune-Mediated Diseases

Despite the broad heterogeneity of autoimmune and autoinflammatory diseases’ etiology, several recurrent observations on phenotypical and functional alterations of MAIT cells were made. In the peripheral blood of patients diagnosed with different disorders {systemic lupus erythematosus [67], ankylosing spondylitis [68], rheumatoid arthritis (RA) [69], vasculitis [70], systemic sclerosis [71], primary Sjögren syndrome (pSS) [72], primary sclerosing cholangitis (PSC) [73] and inflammatory bowel disease [31,74], MAIT cells were shown to be significantly reduced. Of note, in some diseases, iNKT cell counts are also decreased, pointing towards a functional interdependence between the populations in these diseases. In Lupus, a strong positive correlation between MAIT cell deficiency and disease activity was seen. Interestingly, not all MAIT cell subsets decreased to the same extent. For instance, an alteration in population composition in terms of the expression of CD4 and CD8 coreceptors was observed in RA and pSS patients [72]. Differences in cytokine secretion profiles of peripheral blood MAIT cells were seen between autoimmune disease patients and matched healthy controls, which could reflect shifts in MAIT cell subsets upon disease development and/or progression. Latest research confirmed that MAIT cell activity is highly dependent on early-life environmental stimulation, including host–microbiome interactions, factors largely postulated to be of relevance to autoimmune disease pathogenesis.

MAIT cells have also been found to infiltrate the tissues affected in the course of immune-mediated diseases. In the synovium of RA and spondyloarthritis patients, MAIT cells accumulate and have distinctively downregulated activation markers, which is consistent with hyporesponsiveness to potent microbial stimuli [69]. The same functional exhaustion was described for MAIT cells infiltrating biliary ducts in primary sclerosing cholangitis, salivary glands of pSS patients [71], and intestinal lesions in the course of inflammatory bowel disease [31,74], showing increased cell turnover (measured by Ki67 stainings) in the latter condition. This suggests that the infiltrating MAIT cells are chronically stimulated, possibly both by a multitude of locally produced inflammatory cytokines and/or TCR-mediated signals. Indeed, loss of mucosal barriers integrity—commonly associated with autoimmune diseases—may result in an increased exposure to microbial stimuli, which might lead to upheld activation and, ultimately, exhaustion of MAIT cells. In conclusion, these findings suggest a significant role of MAIT cells in the course of autoimmune pathologies.

## 4. MAIT Cells in Graft-Versus-Host Disease

The role of MAIT cells in the reconstitution of the immune repertoire after allogenic hematopoietic stem cell transplantation has been investigated in several studies. The recovery of MAIT cells in the graft recipient was found to be dependent on donor cells transferred directly or as precursor cells, and their proliferation correlated with the abundance of intestinal commensal bacterial species [75]. Reconstituting MAIT cells were functionally impaired and sensitive to chemotherapeutics [76]. Because MAIT cells preferentially localize in the organs affected by graft-versus-host disease (GvHD), their role in this immune phenomenon was studied. An increase in the peripheral blood MAIT cells early after stem cell transplantation was associated with reduced risk of GvHD development [75,77]. The authors suggested that this could be due to MAIT cell potency of suppressing CD4+ T cell proliferation. Studies in mice reported that deficiency in MAIT cells aggravates GvHD and reduces survival rates. Further experiments showed that MAIT cell depletion allowed for proliferation of alloreactive donor T cells, especially in the colon [78]. The effect is thought to be IL-17 dependent, having an effect on the microbiota composition, alloantigen presentation, and maintenance of the intestinal barrier integrity. This is in line with previous finding on an IL-17-producing T cell population in GvHD [79]. Of note, granulocyte colony-stimulating factor (G-CDF), routinely used for stem cell mobilization in donors, was found to promote IL-17 secretion by circulating donor CD8+ MAIT cells [80]. This has to be taken in consideration given that IL-17 has a dual role in intestinal homeostasis [81].

## 5. MAIT Cells in Malignant Diseases

Recent research sheds light on the major role of microbiome in the development of the host’s immune system as well as in infectious, inflammatory, and malignant diseases. Moreover, infectious agents are estimated to trigger the development of 20% of cancers worldwide [82]. Given their abundance in humans, the ability to rapidly secrete a plethora of inflammation mediators, and the link with microbiota, the investigation of MAIT cells is gaining interest in the oncology field, and the distinctive characteristics of MR1 restricted antigen presentation further urge researchers to determine their functional role in tumorigenesis and cancer immunity. Since the first discovery of riboflavin-related antigens as MAIT cells-activating MR1 ligands, the list of other MR1 targets expanded significantly. Folate-derivatives formylpterins were ascertained as MAIT cells inhibitors. Non-vitamin B-derived ligands were found to bind to MR1 as well, including pharmacological agents and their metabolites: diclofenac, 3-formylsalicylic acid, and intermediate methotrexate metabolite [83]. Thus far, no endogenous ligands have been identified, but their existence is supported by the persistence of MAIT cells selection in the thymus of germ-free mice and the minimal surface expression of MR1 protein by certain human cell lines [2,18]. The identification of endogenous MR1 ligands would greatly augment our understanding of the role of MAIT cells in autoimmune diseases and tumor immunity. Concurrence of immune phenomena among autoimmune and malignant diseases often allows for approximate translation of findings, and this paradigm also applies to MAIT cell biology. The rapidly growing understanding of their function and the promise of its modulation might potentially put MAIT cells at the center of modern immunotherapy development.

### 5.1. Hepatic Malignancies

**Figure 2:**
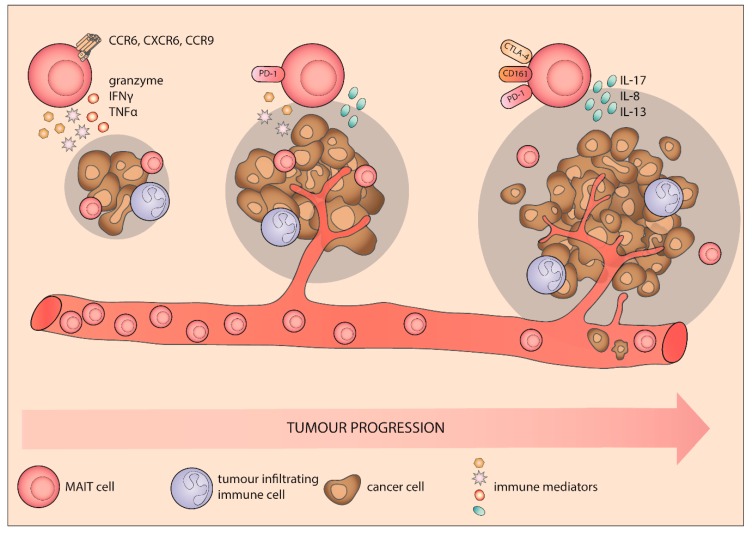
MAIT cells in solid tumors. The frequency of MAIT cells in the peripheral blood negatively reflects the progression of the studied malignant diseases. The reduction in circulating MAIT cells coincided with the overexpression of immune exhaustion markers on their surface. By their tissue-homing properties, MAIT cells actively infiltrate solid tumors and metastatic lesions. However, MAIT cells isolated from the tumor microenvironment seem functionally altered, as they show a reduced capacity to produce granzyme, IFNγ, and TNFα and more IL-17, IL-8, and IL-13. MAIT, mucosal-associated invariant T cell; IFNγ, interferon gamma; TNFα, tumor necrosis factor alpha, IL-17, interleukin 17; CCR6, C-C chemokine receptor 6; CXCR6, C-X-C motif chemokine receptor 6; CCR9, C-C chemokine receptor type 9.

The liver is an immunologically intriguing organ continuously exposed to microbial and food antigens carried by the portal vein circulation and densely populated by unusual immune cells, particularly MAIT and iNKT cells [84,85]. Despite this notable immune surveillance, malignant transformation is a relatively common complication of chronic liver inflammation in both autoimmune disorders and persistent viral infections. Therefore, elucidating the role of MAIT cells in different chronic liver diseases is crucial in understanding processes leading from sustained inflammation to hepatic cancer development (Figure 2).

In hepatocellular carcinoma (HCC) patients, peripheral blood MAIT cells are numerically and functionally depleted [86]. Similar discoveries were made in terms of several chronic inflammatory conditions that are associated with an increased risk of malignant transformation in the liver, namely hepatitis C virus (HCV) infection [87], primary biliary cholangitis [88], and alcoholic and non-alcoholic fatty liver disease [89]. The degree of MAIT cell deficiency corresponded with disease progression, measured as HCC advancement, extent of liver fibrosis, or serum level of liver damage markers. The remaining circulating MAIT cells showed an upregulated expression of activation and exhaustion markers: immune checkpoint molecules such as PD-1 in HCC, and CD38, CD39, CD69, and HLA-DR in autoimmune liver disease and non-alcoholic fatty liver disease. This was reflected by decreased production of IFN-γ. Interestingly, peripheral blood MAIT cells from patients with chronic liver inflammation selectively maintained some of their secretory function. While incapable of upregulating granzyme production upon stimulation, MAIT cells were still able to produce IL-17 [90]. This preferential maintenance of IL-17 production in exhausted MAIT cells could be significant in the context of cancer development and liver fibrosis [91,92]. MAIT cells were recently shown to contribute to liver fibrosis progression by two mechanisms: through activation of hepatic stellate cells via IL-17 [90] and through MR1 signaling-dependent enhancement of hepatic myofibroblast proliferation [93].

MAIT cells express a repertoire of chemokine receptors associated with liver (and mucosal tissue) homing capacities. The peripheral blood MAIT cells of HCC patients expressed CCR6, CXCR6, and CCR9, but these molecules were decreased on their liver cell counterparts and were even further reduced near the tumor burden [86]. The density of MAIT cells in the liver was significantly decreased in the tumor compartment compared to peritumor tissue and non-affected liver [94]. A transcriptomic analysis of MAIT cells in healthy liver tissue, peritumor, and tumor microenvironment revealed striking phenotypical differences. The differentially regulated genes were mostly related to metabolism, cytokine secretion, and cytolysis, pointing towards the capacity of tumor microenvironment to modulate MAIT cell metabolism and effector function [86]. Studies of MAIT cells in hepatic metastases are in line with these findings, showing that MAIT cells could infiltrate metastatic foci but became dysfunctional [95]. Tumor-infiltrating liver MAIT cells showing an exhausted memory effector phenotype accordingly failed to produce IFNγ and cytolytic molecules. Moreover, they produced IL-8, a cytokine implicated in tumor angiogenesis and progression, which might reflect shifts towards tumor-supportive MAIT cells phenotypes at metastatic sites [86].

Persistent infections with hepatotropic viruses are the leading cause of HCC [96], and a drop in circulating MAIT cells was observed for both HCV and hepatitis B virus (HBV) patients [97,98]. The depletion correlated with signs of immune senescence and exhaustion of conventional T cell populations, pointing towards a close relationship between the function of MAIT cells and the immune environment. Strikingly, MAIT cells of chronic HBV patients frequently expressed the surface molecule CD57 [99], which is associated with immunosenescence [100]. Furthermore, they expressed upregulated levels of exhaustion markers PD-1 and CTLA-4, which reflected functional impairment of perforin, granzyme, and IFN-γ production [99]. Superinfection with hepatitis D virus is known to exacerbate chronic HBV-related hepatitis and increase the risk of developing cancer [101]. Recently, immune alterations in hepatitis D virus patients were shown with a dramatic depletion of circulating MAIT cells and functional impairment of the liver-resident subset, particularly in response to MR1-specific ligands [102]. It might be possible that the loss of a functional (protective) MAIT cell population occurs upon progression of hepatotropic virus-related carcinogenesis.

### 5.2. Mucosal-Associated Cancers

In studies that included patients with various malignant tumors, a deficiency in circulating MAIT cells was observed only for mucosal-associated cancers, namely gastric, colon [58], and lung cancer [59]. Moreover, this MAIT cell depletion correlated with the stage of the disease defined by the N parameter, the levels of serum carcinoembryonic antigen (CEA), and the patient hemoglobin. Furthermore, the composition of MAIT cell subsets in the peripheral blood of colon cancer patients were different from the matched healthy control group [59]. The subset of CD8+ memory MAIT cells was particularly reduced [58].

In colon cancer patients, MAIT cells were found to infiltrate tumor tissue [32,58,59,103,104]. This is in accordance with the pattern of tissue-homing chemokine receptors of the MAIT cells isolated from the peripheral blood and the intestinal mucosa of colon cancer [59]. qPCR analyses showed that their absence correlated with IFN-γ (decreased) and IL-17A (increased) detection in tumor tissues. Accordingly, circulating MAIT cells of colon cancer patients produced higher levels of IL-17 compared to the healthy control group [58], suggesting a shift towards Th17 phenotype in MAIT cells of colon cancer patients. In vitro studies on peripheral blood MAIT cells isolated from healthy individuals demonstrated that they possess lymphokine-activated killer activity and direct cytotoxicity to myelogenous leukemia cells after activation [59]. Coincubation assays with colorectal carcinoma cells further showed that activated MAIT cells can induce cell cycle arrest in malignant cells in a contact- and dose-dependent manner. Moreover, similar coculture experiments led to a decreased viability of an esophageal adenocarcinoma cell line [105]. These findings prove that MAIT cells are equipped and have the potential to oppose carcinogenesis, at least in vitro. The degree of MAIT cell infiltration into the malignant tissue, however, correlated with disease advancement [58] and poor prognosis [103]. The functional assessment of tumor-infiltrating MAIT cells revealed an enrichment in IL-17-producing and a depletion in IFN-γ-producing subpopulations compared to the unaffected colonic tissue of the same patients [58]. Furthermore, chronic stimulation of MAIT cells results in an increase of IL-13 gene expression and consequent IL-13 production [32]. This finding is particularly interesting in the context of high expression of IL-13 receptor on precancerous intestinal polyps and colorectal cancer tissue [32]. MAIT cell immune exhaustion is likely dependent on soluble mediators released by the tumor mass, as stimulating peripheral blood mononuclear cells with tumor conditioned medium resulted in a decrease in Th1 cytokines [105]. Determining the exact factors causing a switch from anti-tumor to tumor-favorable MAIT cell phenotype would be key for the design of future immunotherapeutics.

### 5.3. Non Mucosal-Associated Solid Tumors

The functional impact on MAIT cells, and hence their anti-tumor activity, seems to be slightly different in the context of non-mucosal associated solid tumors (such as brain, kidney, thyroid, and breast cancer). Peripheral blood MAIT frequencies were higher in non-mucosal associated solid tumors [59]. An early study of invariant TCRs expression in solid brain and kidney tumor tissues revealed clonal expansion of MAIT cells paired with iNKT cell infiltration and correlating with proinflammatory cytokines expression [106].

### 5.4. Haematological Malignancies (Multiple Myeloma)

To date, little is known about MAIT cell function in hematological malignancies. Multiple myeloma (MM) cell lines were shown to express the MR1 protein, capable of binding and presenting vitamin B metabolites to MAIT cells. In response, MAIT cells isolated from healthy middle-aged donors specifically lysed these myeloma cells in vitro, with efficiency and kinetics similar to natural killer (NK) cells [62]. Nevertheless, MAIT cell frequencies were greatly diminished in untreated MM patients and particularly the CD8+ and the CD8-CD4- subsets were affected [47]. Effector functions of MM patients MAIT cells were further decreased, with fewer IFNγ- (and TNFα)-producing cells, although IFNγ-producing capacity seemed restored in relapsed/refractory MM patient samples [47,62]. Favreau et al. also showed increased PD-1 levels on both circulating MAIT and iNKT cells [47]. In vitro blockade of the PD-1 receptor resulted in restoration of MAIT cell function and enabled reciprocal activation of the two innate-like cell populations [47]. These results point towards the potential of MAIT cells as immunotherapeutic target in MM, both directly for their antitumor immunity by direct cytolytic activity and indirectly by stimulating a range of innate and adaptive immune cells.

### 5.5. A Role for MAIT Cells in Cancer Immunotherapy?

The tumor microenvironment is created by the malignant cells as well as the infiltrating immune cells, together generating complex and multidirectional stimuli. Because of their secretory repertoire and the ability to activate and steer other immune cells, MAIT and iNKT cells are postulated to strongly contribute to the tumor immunosurveillance. Interestingly, iNKT cells have been explored as targets in cancer therapy, primarily because of their strong anti-tumor capacities, their ability to functionally modulate other immune cells, and the expected lack of allogeneic (anti-host MHC) responses—features they share with MAIT cells. Consequently, similar strategies might be applied with regard to MAIT cells and, conceivably, valuable lessons can be learned from published iNKT cell-based trials. The use of α-GalCer and other glycolipids to activate iNKT cells has engendered preclinical success in mice, leading to multiple clinical trials with small but significant cohorts of patients with adenocarcinoma (AC), large-cell carcinoma (LCC), multiple myeloma (MM), non-small-cell lung cancer (NSCLC), renal cell carcinoma (RCC), or squamous cell carcinoma (SCC) [107]. Although the translation of these preclinical benefits into clinical trials is associated with some challenges, they provided encouraging results with tumor stabilization and, in some cases, tumor regression. Similarly, MAIT cell activation strategies with agonist ligands such as VitB ligands might be of potential interest. Notably, 5-OP-RU seems to mainly influence MAIT17 cell thymic development [18], which could be of concern given the cancer-promoting activity of MAIT cell-secreted IL-17. A broader range of molecules, including drugs and drug-like molecules and potentially (still to be identified) endogenous ligands could be added to these attempts [83]. Whether MAIT cells can recognize tumor neoantigens in the context of MR1 presentation and whether they can respond to tumor cells are important questions. In this regard, a recent in vivo study by Yan et al. unexpectedly showed that MAIT cells were found to promote cancer metastasis by suppressing T and/or NK cells (which was partly IL-17 dependent) after interaction with MR1 molecules expressed on tumor cells and pre-treatment of tumor cells with 5-OP-RU increased lung metastases [9]. These data suggest that blocking MR1 (or providing inhibitory MAIT ligands) may represent an interesting strategy for cancer immunotherapy [9]. In contrast, another recent report shows that a human T cell clone that recognizes an MR1 restricted nonbacterial antigen (potentially a cancer-specific or associated metabolite) mediates potent lysis of different cancer cell types [108]. In addition, other atypical MR1 restricted T cells (not responding to 5-OP-RU) have been described [16,19,109], some reacting towards tumor cells such as “MR1T cells” [8], but more research is needed to unravel the precise nature and function of these cells. These findings seem to be related to some extent to findings in the NKT cell field, where CD1d restricted type 2 NKT cells that—in contrast to iNKT cells (also called type 1 NKT cells), show a diverse (broad) TCR repertoire and ligand recognition (not responding to αGalCer)—have been associated with the regulation and the inhibition of tumor immunity in murine tumor models and patients (reviewed in [110]). Altogether, this warrants further investigation into the role and the antigen reactivity of diverse MAIT (-like) cell subsets in the context of anti-tumor responses.

An important hurdle for targeting iNKT cells is their low and variable frequency in peripheral circulation. MAIT cells are far more prevalent in humans than iNKT cells, although they might also be reduced under malignant conditions. Adoptive iNKT cell therapies making use of chimeric antigen receptors (CAR-iNKT) engineering can circumvent this issue and seem to provide promising results with prolonged in vivo persistence and superior target-specific killing activity in preclinical studies [64,111,112]. Given their restricted TCR usage, this strategy can also be explored for MAIT cells (MAIT-CAR). Another important obstacle is the observation of functional exhaustion of iNKT, which has also been shown for MAIT cells [113,114]. For instance, administration of soluble iNKT ligands have been linked to the upregulation of inhibitory co-stimulatory molecules such as PD-1 [115]. A combination therapy with a MAIT cell stimulus and an immune checkpoint inhibitor might therefore be needed for optimal and long-term antitumor responses [47]. Development of diagnostic tools that enable appropriate patient stratification and selection will be the main challenge. Together with response to treatment biomarker identification, these will allow one to maximize treatment efficacy and reduce the socio-economic burden currently associated with immunotherapies.

## 6. Conclusions

MAIT cells are a unique, evolutionarily conserved population bridging innate and adaptive immunity. The development of MAIT cells is strongly dependent on commensal microbiota stimuli; accordingly, the population is enriched at mucosal sites [3]. This underlies a reciprocal interaction, as MAIT cells are capable of shaping the microbial population as determined in MR1 deficient mice [78]. They recognize a range of pathogens upon a shared metabolic signature, and their TCR is restricted to the recognition of non-protein antigens presented by non-polymorphic MR1 proteins. Recent studies have shed light on fascinating features of MAIT cell biology and their functional regulation. Depending on the co-stimulating factors and the inflammatory environment, MAIT cells can exert different roles [29], which is of relevance in disease context. Moreover, via direct contact and copious production of soluble mediators, MAIT cells further influence other cell populations and orchestrate the immune response.

MAIT cells have been linked to a variety of diseases and pathological conditions, including infectious and autoimmune disorders and cancer. Studies on MAIT cell function in autoimmune diseases could be of particular relevance to cancer research, since multiple paraneoplastic syndromes resemble the symptoms of autoimmune diseases. Furthermore, malignant transformation is a late but frequent complication of many autoimmune diseases. For example, lifetime incidence of cholangiocarcinoma is as high as 20% among primary sclerosing cholangitis patients, and the presence of abdominal malignancies has been strongly linked to the mortality rate in these patients [116]. pSS disease is associated with a 16-fold increased risk of developing non-Hodgkin’s lymphomas [110,117]. Sarcoidosis, perceived as a manifestation of chronic inflammation, is also associated with increased risk of cancer [118]. Persistent inflammation causes DNA damage and increases tissues’ susceptibility to oncogenesis [119]. MAIT cells were found to infiltrate chronically inflamed tissues, in which they displayed a hyperactivated and exhausted phenotype [120]. However, their abundance in solid tumors and peritumor zones is much lower compared to the healthy tissue. Tumor-infiltrating MAIT cells are functionally distorted, which is reflected by the production of potentially tumor-promoting cytokines. Singular discrepancies in the results of studies on MAIT cells in cancer might be the consequence of large interindividual differences, disease heterogeneity, small study populations, and MAIT cell identification and characterization techniques. MR1 molecule tetramers only recently became commercially available and, in the majority of thus-far published studies, MAIT cells were identified as TCRVα7.2 and CD161 positive cells. Therefore, future work with these advanced tools and new research techniques—such as single-cell RNA sequencing—will shed further light on the phenotype and the function of MAIT and other MR1 restricted cells in the context of malignancies and their potential as targets in immunotherapy.

## Figures and Tables

**Table 1:** MAIT and invariant natural killer T cells (iNKT) cell characteristics and roles in malignancies.

Feature	MAIT Cells	iNKT Cells	Ref.
TCR combinations	Human: TRAV1-2–TRAJ33 (pairs predominantly with TRBV20-1 or TRBV6 (polyclonal); TRAJ12 and TRAJ20 have also been demonstrated Mice: Vα19 combined with Vβ6 or Vβ8	Human: TRAV10–TRAJ18 (invariant) pairs predominantly with TRBV25-1 (polyclonal) Mice: Vα14–Jα18 combined with Vβ2, Vβ7, or Vβ8.2	[25,41]
Prevalence in peripheral blood	1–10% of T lymphocytes in peripheral blood of a healthy adult (mouse 0.1%); increases until the 3rd decade of life and then drops	0.01–1% of T lymphocytes in peripheral blood of a healthy adult; decreases with age	[13,48,49]
Tissue frequency	40–50% of intrahepatic T lymphocytes (in contrast, 0.6% in mice) 1–5% of lymphoid cells in the intestinal mucosa 2% of skin T cells, mostly residing at the interface of dermis and epidermis	1% of intrahepatic lymphocytes (in contrast 10–30% in mice) 5% of lung-resident lymphocytes >15% of adipose tissue T cells	[40,50,51,52,53]
MHC restriction	MR1	CD1d	[48]
Activating ligands	Riboflavin intermediate metabolites (e.g., 5-OP-RU and 5-OE-RU) Drug metabolites (e.g., diclofenac metabolites).	Bacterial and synthetic glycol(sphingo)lipids and phospholipids (e.g., α- GalCer, β-GlcCer, diacylglycerols, cholesteryl α-glucosides and ether-bonded mono-alkyl glycerophosphates)	[7,54]
Inhibiting antigens/ligands	Folate derivatives (e.g., 6-FP, Ac-6-FP),	GM2 ganglioside, aminobisphosphonates	[55,56]
Cell markers and phenotype	PLZF, RORγt, T-bet; IL-7R, IL-12R, IL-15R, IL-18R; CD3, CD4, CD8, CD161, CD44, CD69, CD25; display an effector-memory phenotype prior to antigen exposure, TCR-independent activation	T-bet, GATA3, PLZF, RORγt; IL-12R, IL-18R, IL-27R; CD3, CD4, CD8, CD161, CD69, CD103, CRTAM; display an effector-memory phenotype prior to antigen exposure, TCR-independent activation	[25,39,50,57]
Subsets	MAIT1, MAIT17	NKT1, NKT2, NKT10 and NKT17; ability to further differentiate functionally under inflammatory conditions	[19,50]
Role in solid tumors	- ↓ of peripheral blood frequency - cytotoxicity towards malignant cells in experimental conditions - infiltrate tumor environment; express exhausted phenotype and can produce cytokines supporting cancer growth - unestablished, equivocal role in tumor progression and metastasis	- recognize tumor neoantigens - decelerate tumor growth in experimental model of cancer development - exhibit cytotoxicity against cancer cells in experimental settings - deleterious role in lung cancer? - direct and indirect immunosurveillance	[15,32,47,58,59,60,61]
Role in hematological malignancies	- immune checkpoint therapy counters MAIT cell deficiency in peripheral blood - direct cytotoxicity against multiple myeloma cells expressing MR1 molecule in experimental settings	- effective coaddition of iNKT cell stimulation to standard therapies in multiple myeloma and lymphomas	[47,62,63,64]
Dependence on microbiome	Commensal microbiota required for MAIT cell maturation, activation of MAIT cells by microbial antigens	(immature) iNKT cell numbers increase in germ-free mice, microbial antigens induce proinflammatory responses in iNKT cells	[65,66]

MAIT, mucosal-associated invariant T cells; iNKT, invariant natural killer T cells; TCR, T cell receptor; MR1, major histocompatibility complex class I-like protein; 5-OP-RU, 5-(2-oxopropylideneamino)-6-D-ribitylaminouracil; 5-OE-RU, 5-(2-oxoethylideneamino)-6-D- ribitylaminouracil; α- GalCer, alpha-Galactosylceramide; β-GlcCer, beta-glucosylceramide; Ac-6-FP, acetyl-6-formylpterin; PLZF, promyelocytic leukaemia zinc finger protein; RORγt, Retineic-acid-receptor-related orphan nuclear receptor gamma; T-bet, T-box transcription factor; GATA3, GATA binding protein 3; IL-7R, interleukin 7 receptor; CRTAM, cytotoxic and regulatory T cell molecule.
